# Quantification of Unintegrated HIV-1 DNA at the Single Cell Level *In Vivo*

**DOI:** 10.1371/journal.pone.0036246

**Published:** 2012-05-04

**Authors:** Rodolphe Suspène, Andreas Meyerhans

**Affiliations:** Department of Virology, Institute of Medical Microbiology, University of the Saarland, Homburg/Saar, Germany; George Mason University, United States of America

## Abstract

In the nucleus of HIV-1 infected cells, unintegrated HIV-1 DNA molecules exist in the form of one and two LTR circles and linear molecules with degraded extremities. In tissue culture they are invariably more numerous than the provirus, the relative proportion of integrated to unintegrated forms varies widely from ∼1∶1 to 1∶10 and even over 1∶100. *In vivo*, this ratio is unknown. To determine it, single nuclei from two infected patients with a known provirus copy number were microdissected, HIV DNA was amplified by nested PCR, cloned and individual clones sequenced. Given the extraordinary sequence complexity, we made the assumption that the total number of distinct sequences approximated to real number of amplifiable HIV-1 DNA templates in the nucleus. We found that the number of unintegrated DNA molecules increased linearly with the proviral copy number there being on average 86 unintegrated molecules per provirus.

## Introduction

The phenomenal intrapatient variation of human immunodeficiency virus type 1 (HIV-1) genome needs no introduction [1], [2], [3], [4], [5], [6], [7]. The absence of proofreading mechanisms associated with the reverse transcriptase, the high recombination rate and high turnover are the main factors [8], [9], [10]. Whenever multiple infections occur, recombination invariably follows. Recombination is present at all levels of HIV genetics [11], [12]. Within an infected individual recombinant genomes show up in network analyses of HIV sequences [13], [14], [15], [16]. In an animal model, macaques inoculated simultaneously with SIVmac239Δ*vpx* or Δ*vpr* and with SIVmac239Δ*nef*, the emergence of wild-type virus was detected in blood in as little as 2 weeks post-inoculation [17], [18]. Finally, some strains in widespread circulation are clearly composites of at least 2–3 other clades [19], [20], [21], [22].

Recently, it was shown that in infected patients ∼85% of infected CD4^+^ T cells in blood contain only one copy of HIV-1 DNA [23]. This would suggest a limited potential for recombination in virus produced by these cells. However, HIV replicates mainly in secondary lymphoid organs. The spleen is a secondary lymphoid organ replete with white pulps and germinal centres and it is in such structures that HIV recombination will occur. Indeed, the majority of HIV-1 infected cells *in vivo* harbour multiple proviruses and additional unintegrated DNA molecules [4], [5], the average proviral copy number per infected cell being 3–4 with a range of 1–8 [5]. Greater than 75% of infected cells harboured two or more proviruses [5]. The ratio of unintegrated/provirus DNA molecules was not addressed [5]. It is well known that proviruses are accompanied by unintegrated DNA forms, either as covalently closed circles with one or two LTRs or linear molecules with ends degraded by exonucleases [24], [25], [26], [27]. In tissue culture experiments the relative proportion of integrated to unintegrated forms varies widely from ∼1∶1 to 1∶10 and even over 1∶100 [28], [29].

How can this ratio be addressed for single cells *in vivo*? Fluorescence *in situ* hybridization (FISH) can quantify the number of proviruses per cell [5]. In this work, Jung et al., reported extensive genetic variability within the hypervariable V1V2 region of viruses from two patients. Indeed, the vast majority of sequences were unique. This is perhaps not too surprising as the hypervariable V1V2 region of *env* is one of the most variable regions of the HIV-1 genome and thus offers the greatest resolution. This choice also meant there was no interference from the HIV-1Δ*env* probe used for FISH [5]. Given the phenomenal variation for these two patients, it is probably a reasonable assumption to equate the number of unique sequences with total number of distinct molecules within the nucleus. Accordingly, if sequencing was performed on the nuclei of single cells, it is possible to estimate the number of unintegrated DNA copies per nucleus.

## Materials and Methods

### Patients/Ethics Approval

The two patients, B and R, have been already described [1], [4], [5], [30]. Briefly, patient B was at stage clinical B1 and had a blood CD4 count of 583/µl and a plasma viremia titre of 5,900 RNA copies/ml. Patient R was at stage C2, while the blood CD4 count and viremia were 317/µl and 126,000 RNA copies/ml respectively. Splenic tissues from patients B and R came from Hôpital Saint-Louis (Paris, France), [1], [4], [5], [30]. Ethics approval for the two patients have been obtained from Hôpital Saint-Louis (Paris, France). Informed consent were obtained from the patients B and R.

### Preparation of Cells for Fluorescence In situ Hybridization

Frozen spleen cells from HIV-infected patients were thawed and stimulated with 2.5 µg/ml phythaemagglutinin (PHA) (Difko, Detroit, MI. USA) in the presence of 10 µM azidothymidine (AZT) (Sigma, Taufkirchen, Germany) to prevent the virus spread in the cell culture [1], [4], [5], [30]. After two days of culture in RPMI 1640 (Lonza, Velviers, Belgium) with 10% fetal calf serum (Invitrogen, Karlsruhe, Germany), 1% penicillin/streptomycin (Biochrom AG, Berlin, Germany) and IL-2 (100 U/ml; Chiron Behring, Marburg, Germany), the cell nuclei were prepared via incubation with 0.1 M KCl for 30 min at 37°C, fixed with methanol/acetic acid 3∶1 at 4°C and stored at −20°C. The HIV FISH was performed according to our previous HIV protocol [5]. Nuclei with defined HIV provirus numbers were microdissected using a PALM Robot16 MicroBeam, according to the manufacturer’s instructions (PALM Bernried, Germany), and transferred to PCR tubes with the laser pressure catapulting technique.

### PCR Amplification, Cloning and Sequencing

A fragment of the HIV envelope genome (V1V2) was amplified employing a semi-nested procedure. In order to increase sensitivity and specificity, hot start PCR was performed. First round primers were LV15 5′- gccacacatgcctgtgtacccaca and LV13 5′- CTTTAGAATGCGAAAACCAGCCG while primers SK122 and SK123 5′-CTAAAGCCATGTGTAAATTAACC and 5′- TGGCTCAAAGGATACCTTTGGACA were used for the second round. The first and second round of PCR involved standard amplification, the reaction parameters were 95°C for 5 min, followed by 35 cycles (95°C for 30 s, 55°C for 30 s, and 72°C for 30 s) and finally for 10 min at 72°C. The buffer conditions were 2.5 mM MgCl_2_, 50 mM KCl, 10 mM Tris-HCl (pH 8.3), 200 µM of each dNTP, 100 µM of each primer, and 2.5 units of Taq DNA polymerase (Cetus) in a final volume of 50 µl. The equivalent of 0.2 l of the first round reaction is used as template in the second round. Amplification products were purified from agarose (Qiaex II kit, Qiagen, France) and directly cloned into the pCR2.1-TOPO vector included in the TOPO TA Cloning Kit (Invitrogen Corp., San Diego, CA). After transformation of *E. coli* TOP10F’ Blue cells, up to 4000 clones were picked and sequenced by GATC Biotech.

### Determination of PCR-mediated Recombination Frequency

To appreciate the PCR-mediated recombination frequency, ten picograms each of DNA corresponding to V1V2 mini-prep clones 01 and 16 were mixed and subjected to 35 cycles of PCR with primers L1 and L2 and 1/10 of the first PCR was used to amplify for 35 cycles with primers SK122 and SK123 under the same conditions as those described above [2], [5], [31]. The DNA was purified and cloned into TOPO TA cloning site. About 500 colonies were screened with 4 different ^32^P-labeled oligonucleotides (Probes A1 5′ AACACCAATAATAGTAGCAA and A2 5′ TGATACTTCTAGCTATAGC for clone 01 and B1 5′ GTGCACTAATAATAACACC and B2 5′ TATAGGAAATGATACTACTA for clone 16, data not shown). Plaques giving positive hybridization signals with both couple of primers A1+B2 or/and B1+A2 were considered PCR-mediated recombinants. Recombination was confirmed by sequencing and a recombination frequency of 2/500 or ∼4×10^−3^ established.

## Results

### Quantification of Unintegrated HIV-1 DNA

**Figure 1:**
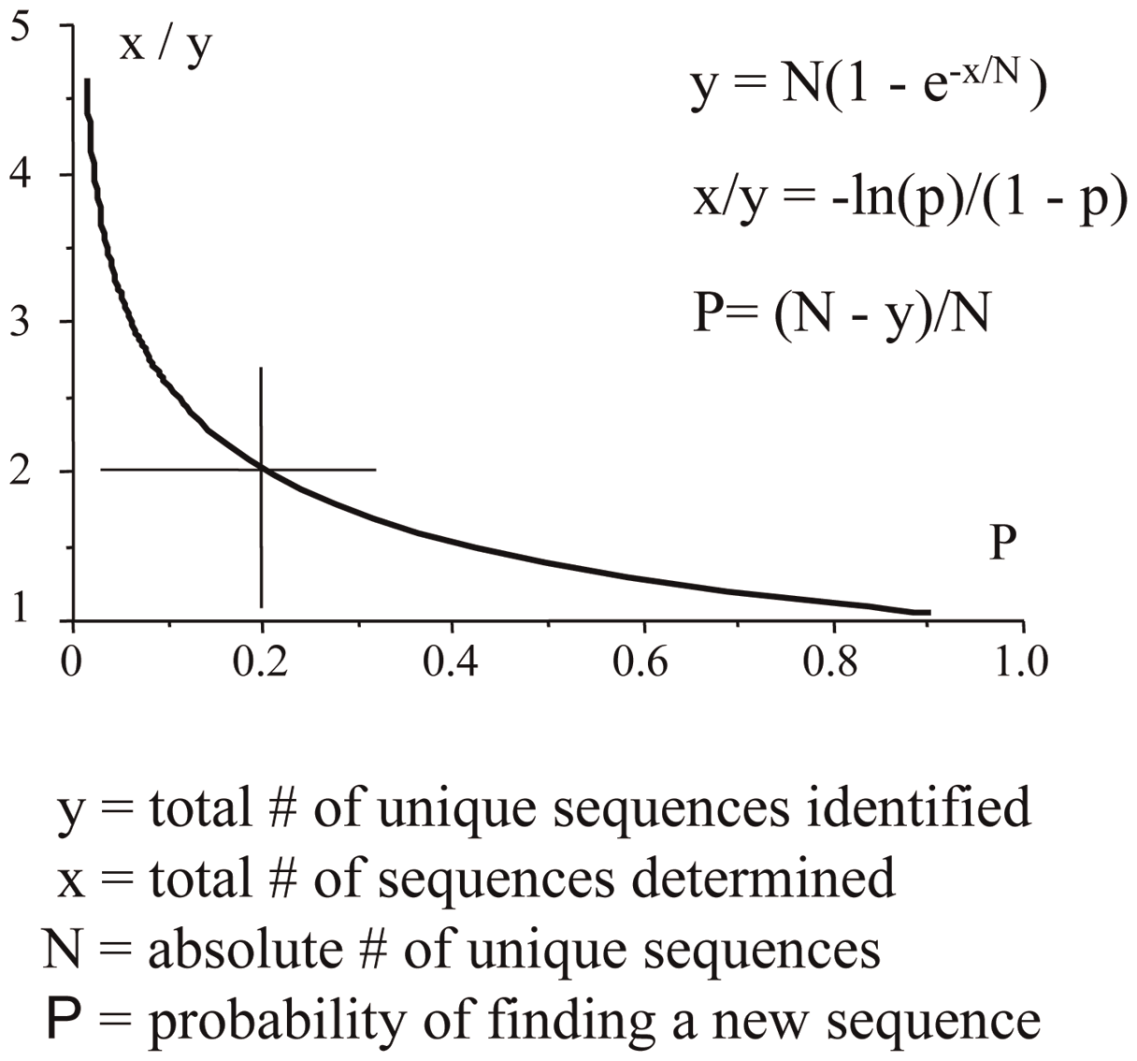
Relationship between the probability P of scoring for a new sequence as a function of the ratio of genetically distinct (y) and total (x) sequences.

**Figure 2:**
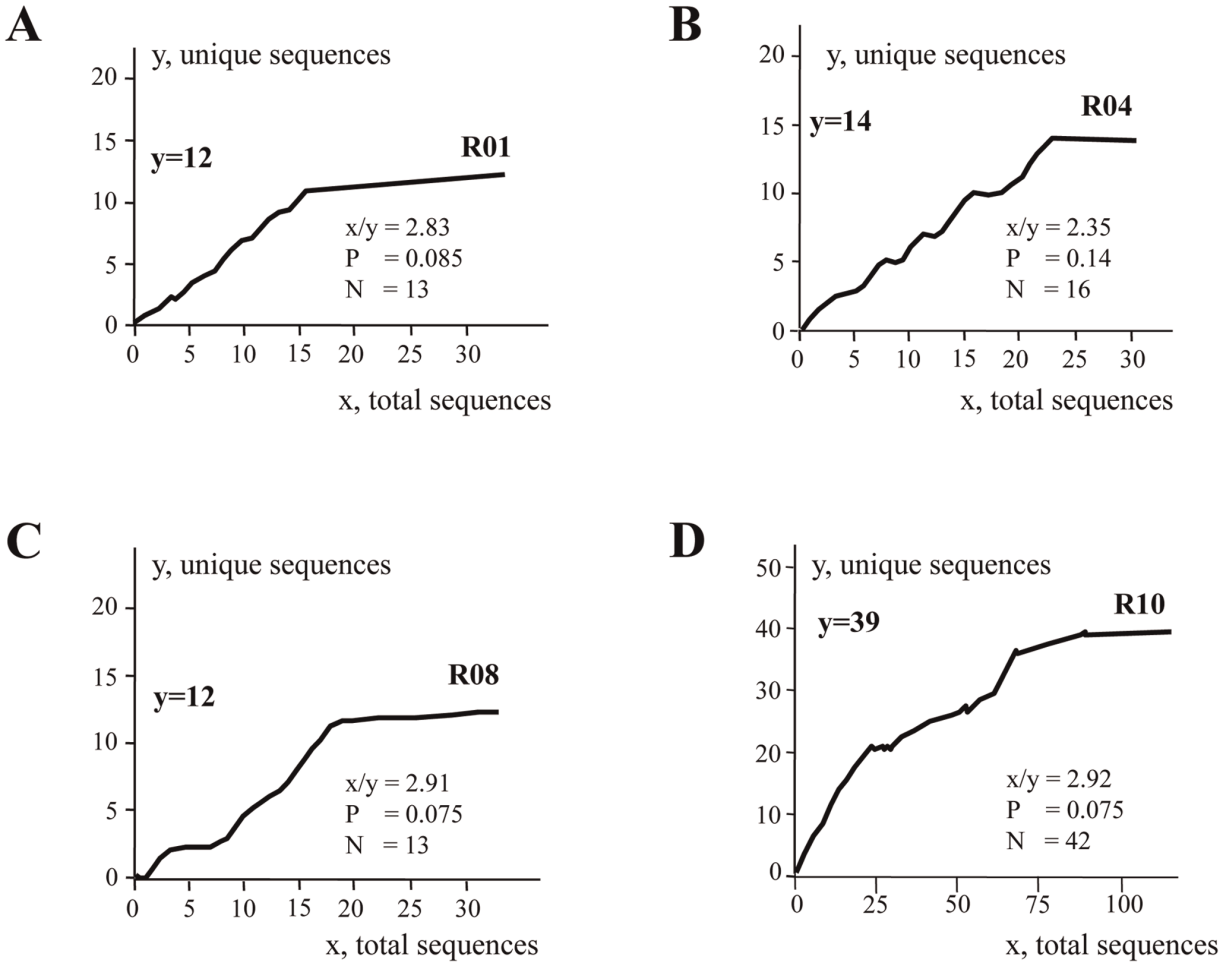
Quantification of genetically distinct HIV-1 V1V2 Env sequences within four splenocytes from patient R harbouring a single HIV-1 provirus.

For sequencing, how many molecules should be sequenced to detect the real number of distinct viral DNA in a sample? We considered P, the probability of finding a new sequence, and N as the absolute number of distinct sequences, while x and y are the number of total and unique sequences experimentally determined respectively. The relationship between P and N is obtained from the equation, P = (N−y)/N (Figure 1). For an x/y ratio ∼2 approximately 80% of unique sequences (y) can be identified. HIV-positive interphase nuclei from two patients (B and R) were laser microdissected and transferred to PCR tubes and the V1V2 *env* region amplified. In order to increase sensitivity and specificity, hot start PCR was performed. Four splenocytes from patient R harbouring a single provirus constituted the starting point (Figure 2). Small numbers of sequences were sufficient for the number of unique sequences (y) to plateau. The values of y ranged from 12–14 to 39, which translates into values of N of 13–16 and 42 respectively (Figures 2). Given the underlying assumption that all genomes are genetically unique, the unintegrated/provirus ratios are ≥13∶1 to 42∶1.

**Figure 3:**
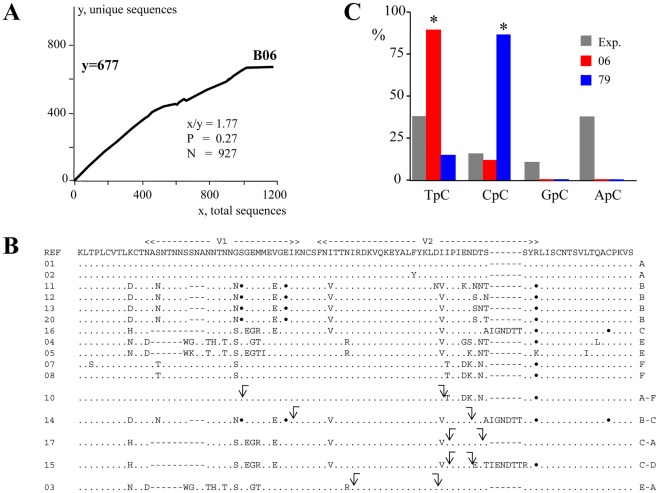
Phenomenal sequence diversity within single cell B06. A) Quantification of unintegrated HIV DNA in a single splenocyte from patient B with 6 proviruses. B) Env V1V2 amino acid sequences from patient B6 are shown and compared to an arbitrarily selected sequence as reference. Only differences are shown. The sequences are coded by capital letters to the right with obvious recombinant molecules indicated. Dots denote synonymous substitutions, while arrows correspond to recombination sites. C) Dinucleotide context associated with two singular APOBEC3 edited genomes. The ordinate represents the substitution frequency as a function of the 5′ nucleotide.

**Figure 4:**
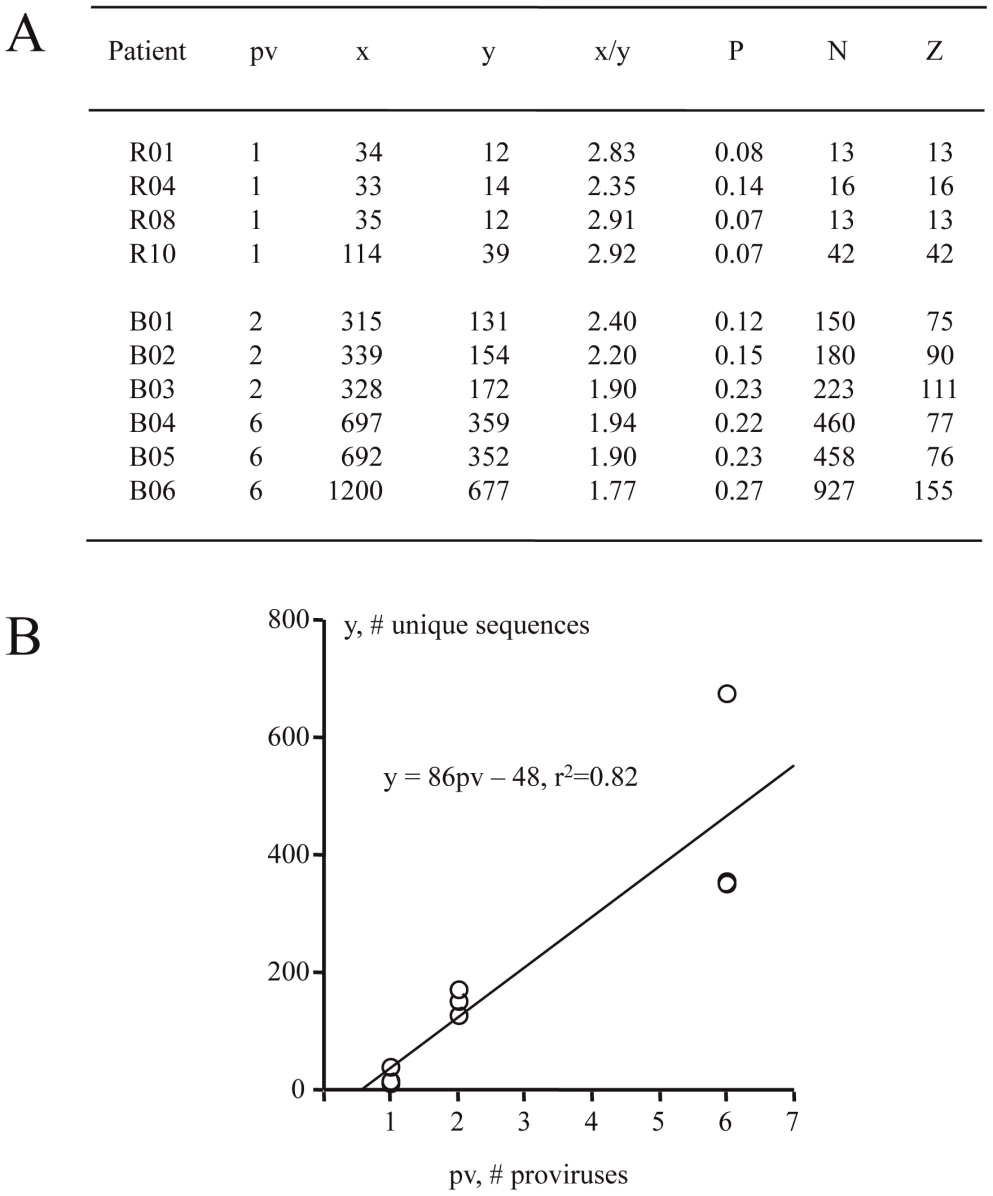
Quantitation of HIV DNA molecules in single nuclei. A) Tabulation of the number of detected and projected unintegrated forms for nuclei from splenocytes of patients R and B. pv, proviral copy number detected by FISH; y, # of unique sequences identified; x, # of sequences determined; P, probability of finding a new unique sequence; N, absolute # of unique sequences; Z, unintegrated DNA/provirus ratio. B) Linear correlation between unintegrated and proviral copy numbers.

A similar analysis was performed on the nuclei of six splenocytes with 2 or 6 proviruses from patient B (Figures 3A and 4A). The number of unique sequences identified ranged from 131 to 172 for cells harbouring 2 proviruses, and between 359 and 677 for those with 6 proviruses (Figure 4A). Values of N ranged from 150–927, while the unintegrated DNA/provirus ratios (Z) were of the order of 75–155 (Figure 4A). Approximately 927 unique HIV genomes were predicted for nucleus B06 (Figures 3A and 4A). A selection of V1V2 protein sequences from B06 is shown in Figure 3B, a number of which are arguably recombinants. Most probably if more sequences from other cells were sequenced it would be possible to identify more recombinants. Indeed, given the frequency of HIV recombination and the 70% fraction of multiply infected splenocytes for patient B, probably all sequences were recombined in a relatively recent time frame. Recombination was confirmed by sequencing and a recombination frequency of 2/500 or ∼4×10^−3^ established. In parallel, Taq polymerase mutation frequency was shown to be f ∼10^−5^, suggesting that the high mutation frequency observed was not associated to Taq polymerase.

### G to A Hypermutation at a Single Cell Level

The B6 nucleus also harboured two G->A hypermutated sequences which were interesting. Such genomes are associated with editing by host cell cytidine deaminases APOBEC3F and 3G in the context of a Δ*vif* genotype. The enzymes show distinct dinucleotide contexts associated with editing, notably TpC>CpC for APOBEC3F and CpC>TpC for APOBEC3G, where the edited base is underlined [32], [33], [34], [35], [36], [37]. The two sequences showed remarkable selectivity for dinucleotide editing context. For clone 06 TpC was very strongly preferred over CpC, while for clone 79 the opposite was apparent (Figure 3C). These striking differences suggest that occasionally only A3F or A3G are packaged, which is feasible given the estimations of low levels packaged into virions [38]. Presumably hypermutated genomes with less startling biases probably reflect co-packaging of both APOBEC3 proteins.

## Discussion

For these two patients, proviruses are accompanied by a very large number of unintegrated forms, varying from Z = 13–155 molecules per provirus (Figure 4A). As only 10 nuclei were analysed (4 from patient R +6 from B), this number is by no means a maximum. Interestingly the relationship between N and pv is linear (Figure 4B). While the sample size of this present study is low and thus has limited statistical power, the results obtained for the patients are similar. Given these large values for Z, it is logical that many splenocytes harbour unintegrated DNA alone. As the present study focussed on cells with FISH-positive proviruses such cells were not scored. While the values for Z are large compared to those derived from tissue culture, a recent report in a very different setting, peripheral blood mononuclear cells from HIV-infected Elite suppressors identified an unintegrated/integrated DNA ratios of between 10/1 to up to 10,000/1 [39].

The extraordinary genetic variation among HIV DNA molecules found in different nuclei indicates that the source of virus infection was very complex. Within an established HIV-infected patient there are three sources of genetically complex virus. These are circulating virus, virus on follicular dendritic cells (FDC) and virus associated with dendritic cells (DC) presented to CD4+ T lymphocytes. As the infection frequency of splenocytes was ∼1%, this rules out circulating virus, as the major source. The virus on FDC surfaces is essentially in the form of immune complexes and is mainly presented to B cells [40]. By contrast DCs present very efficiently HIV to CD4+ T lymphocytes [41]. While the present data cannot distinguish between the FDC and DC sources, the cellular immunology of DCs pleads in favour of the latter.

Using cell free virus to infect cells *ex vivo*, treatment of the target cell by proteasome inhibitors enhances viral growth ∼3 fold indicating that more viruses infect and enter a cell than make it to the nucleus [42]. Assuming a comparable phenomenon *in vivo* the number of virions needed to infect a single splenocyte would be of the order of 40–2800. If other catabolic pathways were operative, the number of virions would necessarily be even greater.

Infection of a single cell by many virions could well be a general phenomenon in virology. However, in order to quantify the number of incoming virions some trait is needed to distinguish them from the replication template for progeny virus. For retroviruses the incoming genome is RNA while the template for transcription is DNA. For RNA viruses, such as poliovirus, there is no such trait distinguishing parent and daughter genomes. Of course recombinants are a tell tale sign. Following vaccination with the three attenuated polioviruses, recombinants among them have been described, as have recombinants between vaccinating strains and wild type poliovirus and other enteroviruses [43], [44]. Although negative stranded viruses such as influenza are known to recombine rarely, recombinants can be identified [45]. Along with the above data such examples indicate that multi-infection is probably commonplace, an inevitable consequence of the capacity of a cell to produce hundreds to thousands of virions in a very small space.

## References

[1] Cheynier R, Henrichwark S, Hadida F, Pelletier E, Oksenhendler E (1994). HIV and T cell expansion in splenic white pulps is accompanied by infiltration of HIV-specific cytotoxic T lymphocytes.. Cell.

[2] Delassus S, Cheynier R, Wain-Hobson S (1992). Nonhomogeneous distribution of human immunodeficiency virus type 1 proviruses in the spleen.. J Virol.

[3] Delwart EL, Sheppard HW, Walker BD, Goudsmit J, Mullins JI (1994). Human immunodeficiency virus type 1 evolution in vivo tracked by DNA heteroduplex mobility assays.. J Virol.

[4] Gratton S, Cheynier R, Dumaurier MJ, Oksenhendler E, Wain-Hobson S (2000). Highly restricted spread of HIV-1 and multiply infected cells within splenic germinal centers.. Proc Natl Acad Sci USA.

[5] Jung A, Maier R, Vartanian JP, Bocharov G, Jung V (2002). Multiply infected spleen cells in HIV patients.. Nature.

[6] Li Y, Kappes JC, Conway JA, Price RW, Shaw GM (1991). Molecular characterization of human immunodeficiency virus type 1 cloned directly from uncultured human brain tissue: identification of replication-competent and -defective viral genomes.. J Virol.

[7] Meyerhans A, Cheynier R, Albert J, Seth M, Kwok S (1989). Temporal fluctuations in HIV quasispecies in vivo are not reflected by sequential HIV isolations.. Cell.

[8] Ho DD, Neumann AU, Perelson AS, Chen W, Leonard JM (1995). Rapid turnover of plasma virions and CD4 lymphocytes in HIV-1 infection.. Nature.

[9] Pierson T, McArthur J, Siliciano RF (2000). Reservoirs for HIV-1: mechanisms for viral persistence in the presence of antiviral immune responses and antiretroviral therapy.. Annu Rev Immunol.

[10] Wei X, Ghosh SK, Taylor ME, Johnson VA, Emini EA (1995). Viral dynamics in human immunodeficiency virus type 1 infection.. Nature.

[11] McCutchan FE (2000). Understanding the genetic diversity of HIV-1.. Aids.

[12] Robertson DL, Sharp PM, McCutchan FE, Hahn BH (1995). Recombination in HIV-1.. Nature.

[13] Cheynier R, Kils-Hutten L, Meyerhans A, Wain-Hobson S (2001). Insertion/deletion frequencies match those of point mutations in the hypervariable regions of the simian immunodeficiency virus surface envelope gene.. J Gen Virol.

[14] Kils-Hutten L, Cheynier R, Wain-Hobson S, Meyerhans A (2001). Phylogenetic reconstruction of intrapatient evolution of human immunodeficiency virus type 1: predominance of drift and purifying selection.. J Gen Virol.

[15] Plikat U, Nieselt-Struwe K, Meyerhans A (1997). Genetic drift can dominate short-term human immunodeficiency virus type 1 nef quasispecies evolution in vivo.. J Virol.

[16] Wain-Hobson S, Renoux-Elbe C, Vartanian JP, Meyerhans A (2003). Network analysis of human and simian immunodeficiency virus sequence sets reveals massive recombination resulting in shorter pathways.. J Gen Virol.

[17] Kim EY, Busch M, Abel K, Fritts L, Bustamante P (2005). Retroviral recombination in vivo: viral replication patterns and genetic structure of simian immunodeficiency virus (SIV) populations in rhesus macaques after simultaneous or sequential intravaginal inoculation with SIVmac239Deltavpx/Deltavpr and SIVmac239Deltanef.. J Virol.

[18] Wooley DP, Smith RA, Czajak S, Desrosiers RC (1997). Direct demonstration of retroviral recombination in a rhesus monkey.. J Virol.

[19] Brown RJ, Peters PJ, Caron C, Gonzalez-Perez MP, Stones L (2011). Intercompartmental recombination of HIV-1 contributes to env intrahost diversity and modulates viral tropism and sensitivity to entry inhibitors.. J Virol.

[20] Carr JK, Wolfe ND, Torimiro JN, Tamoufe U, Mpoudi-Ngole E (2010). HIV-1 recombinants with multiple parental strains in low-prevalence, remote regions of Cameroon: evolutionary relics?. Retrovirology.

[21] Hoelscher M, Kim B, Maboko L, Mhalu F, von Sonnenburg F (2001). High proportion of unrelated HIV-1 intersubtype recombinants in the Mbeya region of southwest Tanzania.. Aids.

[22] Rousseau CM, Learn GH, Bhattacharya T, Nickle DC, Heckerman D (2007). Extensive intrasubtype recombination in South African human immunodeficiency virus type 1 subtype C infections.. J Virol.

[23] Josefsson L, King MS, Makitalo B, Brannstrom J, Shao W (2011). Majority of CD4+ T cells from peripheral blood of HIV-1-infected individuals contain only one HIV DNA molecule.. Proc Natl Acad Sci USA.

[24] Brown PO, Bowerman B, Varmus HE, Bishop JM (1987). Correct integration of retroviral DNA in vitro.. Cell.

[25] Brown PO, Bowerman B, Varmus HE, Bishop JM (1989). Retroviral integration: structure of the initial covalent product and its precursor, and a role for the viral IN protein.. Proc Natl Acad Sci U S A.

[26] Farnet CM, Haseltine WA (1991). Circularization of human immunodeficiency virus type 1 DNA in vitro.. J Virol.

[27] Fujiwara T, Mizuuchi K (1988). Retroviral DNA integration: structure of an integration intermediate.. Cell.

[28] Bell P, Montaner LJ, Maul GG (2001). Accumulation and intranuclear distribution of unintegrated human immunodeficiency virus type 1 DNA.. J Virol.

[29] Vandegraaff N, Kumar R, Burrell CJ, Li P (2001). Kinetics of human immunodeficiency virus type 1 (HIV) DNA integration in acutely infected cells as determined using a novel assay for detection of integrated HIV DNA.. J Virol.

[30] Hosmalin A, Samri A, Dumaurier MJ, Dudoit Y, Oksenhendler E (2001). HIV-specific effector cytotoxic T lymphocytes and HIV-producing cells colocalize in white pulps and germinal centers from infected patients.. Blood.

[31] Goodenow M, Huet T, Saurin W, Kwok S, Sninsky J (1989). HIV-1 isolates are rapidly evolving quasispecies: evidence for viral mixtures and preferred nucleotide substitutions.. J Acquir Immune Defic Syndr.

[32] Beale RC, Petersen-Mahrt SK, Watt IN, Harris RS, Rada C (2004). Comparison of the differential context-dependence of DNA deamination by APOBEC enzymes: correlation with mutation spectra in vivo.. J Mol Biol.

[33] Bishop KN, Holmes RK, Sheehy AM, Davidson NO, Cho SJ (2004). Cytidine deamination of retroviral DNA by diverse APOBEC proteins.. Curr Biol.

[34] Henry M, Guetard D, Suspene R, Rusniok C, Wain-Hobson S (2009). Genetic editing of HBV DNA by monodomain human APOBEC3 cytidine deaminases and the recombinant nature of APOBEC3G.. PLoS One.

[35] Langlois MA, Beale RC, Conticello SG, Neuberger MS (2005). Mutational comparison of the single-domained APOBEC3C and double-domained APOBEC3F/G anti-retroviral cytidine deaminases provides insight into their DNA target site specificities.. Nucleic Acids Res.

[36] Suspène R, Sommer P, Henry M, Ferris S, Guétard D (2004). APOBEC3G is a single-stranded DNA cytidine deaminase and functions independently of HIV reverse transcriptase.. Nucleic Acids Res.

[37] Vartanian JP, Henry M, Marchio A, Suspène R, Aynaud MM (2010). Massive APOBEC3 editing of hepatitis B viral DNA in cirrhosis.. Plos Pathog.

[38] Xu H, Chertova E, Chen J, Ott DE, Roser JD (2007). Stoichiometry of the antiviral protein APOBEC3G in HIV-1 virions.. Virology.

[39] Graf EH, Mexas AM, Yu JJ, Shaheen F, Liszewski MK (2011). Elite suppressors harbor low levels of integrated HIV DNA and high levels of 2-LTR circular HIV DNA compared to HIV+ patients on and off HAART.. PLoS Pathog.

[40] Dumaurier MJ, Gratton S, Wain-Hobson S, Cheynier R (2005). The majority of human immunodeficiency virus type 1 particles present within splenic germinal centres are produced locally.. J Gen Virol.

[41] Tsunetsugu-Yokota Y, Akagawa K, Kimoto H, Suzuki K, Iwasaki M (1995). Monocyte-derived cultured dendritic cells are susceptible to human immunodeficiency virus infection and transmit virus to resting T cells in the process of nominal antigen presentation.. J Virol.

[42] Schwartz O, Marechal V, Friguet B, Arenzana-Seisdedos F, Heard JM (1998). Antiviral activity of the proteasome on incoming human immunodeficiency virus type 1.. J Virol.

[43] Jegouic S, Joffret ML, Blanchard C, Riquet FB, Perret C (2009). Recombination between polioviruses and co-circulating Coxsackie A viruses: role in the emergence of pathogenic vaccine-derived polioviruses.. PLoS Pathog.

[44] Zhang Y, Zhu S, Yan D, Liu G, Bai R (2010). Natural type 3/type 2 intertypic vaccine-related poliovirus recombinants with the first crossover sites within the VP1 capsid coding region.. PLoS ONE.

[45] Nelson MI, Viboud C, Simonsen L, Bennett RT, Griesemer SB (2008). Multiple reassortment events in the evolutionary history of H1N1 influenza A virus since 1918.. PLoS Pathog.

